# Therapeutic patient education for severe mental disorders: A systematic review

**DOI:** 10.1017/gmh.2024.68

**Published:** 2024-10-10

**Authors:** Ahmed Waqas, Jorge Cesar Correia, Maaz Ahmad, Tooba Nadeem Akhtar, Hafsa Meraj, Ioannis Angelakis, Zoltan Pataky

**Affiliations:** 1 Department of Primary Care & Mental Health, Institute of Population Health, University of Liverpool, Liverpool, UK; 2 Unit of Therapeutic Patient Education, WHO Collaborating Centre, Department of Medicine, University Hospitals of Geneva, Geneva, Switzerland; 3 Faculty Diabetes Centre, University of Geneva, Geneva, Switzerland; 4 Sharif Medical and Dental College, Lahore, Pakistan; 5 School of Psychology, Trinity College Dublin, Dublin, Ireland; 6 Trinity Centre for Global Health, Trinity College Dublin, Dublin, Ireland; 7 Greater Manchester Mental Health NHS Foundation Trust, Manchester, UK

**Keywords:** therapeutic patient education (TPE), psychiatric disorders, systematic review, meta-analysis, depression, psychosis, schizophrenia, multimorbidity

## Abstract

**Objective:**

This systematic review aimed to review therapeutic patient education (TPE) programmes in managing psychiatric disorders, considering the diversity in delivering agents, intervention formats, targeted skills, and therapeutic outcomes.

**Methods:**

Comprehensive database searches, including Web of Science, PubMed, and COCHRANE, were conducted from September 2019 to January 2023, yielding 514 unique records, with 33 making it through rigorous evaluation for full-text review. Eleven studies met the inclusion criteria, focusing on various psychiatric disorders such as depression, bipolar disorder, psychosis, and multiple serious mental illnesses. A total of 38 studies were included from our previous review to supplement the current database search.

**Results:**

TPE programmes exhibited diversity in delivering agents and intervention formats, with a notable presence of multidisciplinary teams and various professionals. The interventions prioritized coping strategies and disease management techniques, though the extent varied based on the disorder. Effectiveness was heterogeneous across studies; some interventions showed significant benefits in areas such as symptom management, coping, and functional improvement, while others reported no significant outcomes.

**Conclusion:**

The findings underscore the potential of TPE in psychiatric care, revealing its multifaceted nature and varied impact. TPE not only addresses deficits but also leverages patients’ existing strengths and capabilities. Despite the reported benefits, a portion of the interventions lacked statistical significance, indicating the necessity for continuous refinement and evaluation.

## Impact statements

This systematic review critically examines therapeutic patient education (TPE) programmes in managing psychiatric disorders, encapsulating a diverse range of delivering agents, intervention formats, targeted skills, and therapeutic outcomes. Key findings demonstrate the integral role of multidisciplinary teams and a variety of professionals in delivering TPE, highlighting its flexible and patient-centric nature. The programmes are shown to significantly enhance coping strategies and disease management skills in patients, contributing to improvements in symptom management, functional abilities, and overall well-being. However, the review also identifies a variation in the effectiveness of different TPE interventions, signalling the necessity for ongoing refinement and rigorous evaluation. The implications of this review are profound for clinical practice, policy-making, and future research in psychiatric care. It underscores TPE’s potential as a comprehensive educational intervention, focusing not just on addressing deficits but also on leveraging patients’ strengths and capabilities. The insights gained can inform the development of more effective, personalized TPE programmes, thus elevating the quality of psychiatric care. Furthermore, this review serves as an invaluable resource for healthcare providers, policymakers, and researchers. It provides a foundation for formulating policies and strategies to enhance the integration and effectiveness of TPE in psychiatric settings, ultimately contributing to better health outcomes and quality of life for individuals with psychiatric disorders.

## Background

Mental illness and psychiatric disorders significantly impact global health, accounting for a considerable proportion of years lived with disability (Vigo et al., 2016). Historically, the trajectory of mental health has shown a troubling rise. In the context of the global disease burden, 25% of the top 20 causes can be attributed to these disorders (Vigo et al., 2016). These disorders are not only health concerns but also have dire social implications. They stand as prominent risk factors for suicide (Moitra et al., 2021). Yet, even against this backdrop, a significant disparity exists in treatment rates (Moitra et al., 2022). This paints a picture of an urgent need for broader treatment coverage, timely intervention, and improved treatment adherence.

Therapeutic patient education (TPE) offers a promising solution to these challenges (Correia et al., 2022a, 2022b). The need to integrate TPE interventions in treating severe psychiatric disorders stems from the significant challenges these conditions pose to individuals and the limitations of traditional healthcare in providing long-term support (Ashworth et al., 2017; Fekadu et al., 2019; Launders et al., 2022). Severe psychiatric disorders require not only immediate treatment but also ongoing management, where patients face daily challenges related to their conditions. TPE, focusing on empowering patients through education and self-management skills, presents an essential supplement to standard treatments (Zhao et al., 2015; Correia et al., 2019). By improving patients’ understanding of their disorders, teaching effective coping mechanisms, and encouraging active participation in their care, TPE can greatly improve life quality for those with severe psychiatric conditions (Correia et al., 2022a, 2022b). This method is in line with the increasing emphasis on patient-centred care in psychiatry, which aims for holistic treatment and recognizes the importance of patients’ involvement in their health management.

Indeed, over the past few decades, TPE has emerged as a transformative approach, evolving with our expanding understanding of mental health (World Health Organization). Very recently, the WHO updated the TPE guide and strengthened the importance of this holistic approach for chronic disease management, independently of the medical specialty (World Health Organization, 2023). In the realm of psychiatric disorders, this education-oriented approach is commonly termed as “psychoeducation.” The National Institute for Clinical and Healthcare Excellence underscores the importance of patient education as a crucial part of early interventions (National Institute for Health and Care Excellence, 2014). Recent research bolsters the case for psychoeducation. Zhao et al. (2015) found improved pharmacological compliance and reduced relapse rates in individuals receiving psychoeducation compared to those under standard care (Zhao et al., 2015; Asher et al., 2017). Delivered by trained healthcare professionals, TPE aims to help patients self-manage their chronic condition over their lifetime, adapting to their evolving circumstances, as well as changes in their condition and treatment (World Health Organization, 2023).

While previous meta-analyses have concentrated on assessing the efficacy of TPE in managing psychiatric disorders (Zhao et al., 2015; Asher et al., 2017), there remains a critical need for a comprehensive investigation that not only evaluates the effectiveness of these interventions but also explores the content and delivery methods. Such an investigation would provide invaluable insights for intervention developers and implementers by highlighting evidence-based strategies that are most beneficial in practice. For instance, a qualitative investigation by Dutra et al. advocates for incorporation of individuals’ intrinsic abilities and capabilities in the strategic planning of care, inclusive of those confronted with mental health adversities (Dutra et al., 2017). This is further emphasized by the NICE, which recommends to cultivate an ecosystem where extant strengths are harnessed and optimized (National Institute for Health and Care Excellence, 2014). This perspective introduces a vital layer to our understanding, advocating for a care environment that actively cultivates and utilizes patients’ strengths.

Given this context, a detailed exploration of TPE’s effectiveness, focusing on intervention content and delivery mechanisms, becomes essential. Such analysis has significant implications for refining TPE intervention manuals, clinical practice guidelines, and enhancing patient outcomes (Correia et al., 2022a, 2022b).

This review is part of a larger project, Putting the pAtient fiRsT: maNagemEnt of chRonic diSeases by tHerapeutIc Patient education (PARTNERSHIP) (Correia et al., 2019; Correia et al., 2022a, 2022b). It builds on our previous meta-analysis which showed that TPE interventions improve several psychiatric and psychosocial outcomes in psychiatric disorders (Correia et al., 2022a, 2022b). Complementing the previous evidence regarding the efficacy of TPE interventions, this systematic review explores the content and delivery of TPE interventions, addressing the following research questions:

1. Which agents are the most effective in delivering these interventions?
2. Which formats and techniques maximize the outcomes of TPE programmes?

## Methods

### Adherence to guidelines

This review is part of a larger project, ‘PARTNERSHIP (putting the patient first: management of chronic diseases by therapeutic patient education)’ (Correia et al., 2019; Correia et al., 2022a, 2022b), which is leading a series of evidence-synthesis studies on the role of TPE in the management of chronic disorders.

This systematic review and meta-analysis adhered to the Preferred Reporting Items for Systematic Reviews and Meta-Analyses (PRISMA) guidelines (Liberati et al., 2009). The protocol for this review was registered in PROSPERO (Correia et al., 2019).

### Search strategy

Our search encompassed the databases of Web of Science, PubMed, and the COCHRANE from September 2019 to January 2023. Table 1 presents the search strategy in detail, with keywords across the concepts relevant to the review. This strategy was informed by our previous meta-analysis from the PARTNERSHIP project (Correia et al., 2019; Correia et al., 2022a, 2022b). We included studies published in English and French without imposing restrictions on the region or publication year. We supplemented this database search by including 38 studies (published until August 2019) from our previously published meta-analysis evaluating effectiveness of TPE interventions across all medical specialties (Correia et al., 2019).

**Table 1.** Search strategy adapted for PubMed

Concept	Terms
Chronicity	((chronic[Title/Abstract] OR “chronic disease”[Title/Abstract] OR long–term[Title/Abstract] OR “chronic disease”[MeSH] OR “chronic disease, hospital”[MeSH])
Psychiatric disorders	(psychiatr*[Title/Abstract] OR mental[Title/Abstract] OR depress*[Title/Abstract] OR anxi*[Title/Abstract] OR ‘substance use’[Title/Abstract] OR ‘substance misuse’[Title/Abstract] OR ‘illicit substance’[Title/Abstract] OR smok*[Title/Abstract] OR alcohol*[Title/Abstract] OR psycho*[Title/Abstract] OR schizophreni*[Title/Abstract] OR bipolar[Title/Abstract])
Outcomes	(“Treatment outcome”[Title/Abstract] OR outcome[Title/Abstract] OR psychosocial[Title/Abstract] OR lab[Title/Abstract] OR “physical outcome”[Title/Abstract] OR stress[Title/Abstract] OR depress*[Title/Abstract] OR “disease recurrence”[Title/Abstract] OR perception[Title/Abstract] OR “Disease progression”[Title/Abstract] OR “self efficacy[Title/Abstract] OR “self management”[Title/Abstract] OR compliance[Title/Abstract] OR adherence[Title/Abstract] OR knowledge[Title/Abstract] OR attitude[Title/Abstract] OR behavior[Title/Abstract] OR “quality of life”[Title/Abstract])
Study design	(trial*[Title] OR RCT[Title] OR ‘randomized controlled’[Title] OR “cluster randomized controlled”[Title] OR intervention[Title] OR “clinical trial”[Publication Type] OR “controlled clinical trial”[Publication Type])
Intervention	AND (“Health education” [MeSH] OR “patient education”[MeSH] OR “Health education”[Title/Abstract] OR “patient education”[Title/Abstract] OR psychoeducation[Title/Abstract] OR “therapeutic education”[Title/Abstract] OR “consumer health information”[Title/Abstract] OR “health knowledge”[Title/Abstract] OR “client education”[Title/Abstract]))

### Operational definitions

The core of this review is to explore TPE programs tailored for psychiatric disorders. We defined TPE interventions as disorder-specific educational interventions, delivered by specialists or trained non-specialist healthcare professionals, designed to enhance the abilities of patients with psychiatric disorders to manage their conditions (Correia et al., 2019; Correia et al., 2022a, 2022b). These are multicomponent interventions ranging from knowledge and self-management techniques to cognitive strategies, and even emotional and experiential components (Correia et al., 2019; Correia et al., 2022a, 2022b).

### Screening process

For the initial screening, two independent reviewers examined titles and abstracts of bibliographic records retrieved. Articles meeting the preliminary criteria underwent full-text examination to finalize their inclusion. In cases of differing opinions between the reviewers, a senior author served as the tiebreaker.

### Inclusion and exclusion criteria

Our inclusion criteria incorporated randomized and cluster-randomized controlled trials that investigated therapeutic education interventions among adults (18 years and older) with psychiatric disorders limited to depressive disorders, bipolar disorders schizophrenia and complex presentations. Non-randomized, quasi-experimental studies, short-form publications, and overlapping datasets were excluded. When encountering multiple research papers derived from the same dataset, we selected the one with the latest follow-up data to prevent data duplication in our analysis.

### Data extraction procedures

Teams of two reviewers (AW, HM, JCC, MA) independently employed a pre-tested Excel-based proforma for data extraction. A pilot extraction, comprising 10% of the studies, was performed independently by both reviewers to ensure inter-rater reliability. The variables considered included rationale for the intervention, setting of intervention, delivery agents, intervention content and dissemination methods.

### Data extraction for intervention content

TPE interventions are multicomponent interventions ranging from knowledge and self-management techniques to cognitive strategies, and even emotional and experiential components. In a prior publication, we delineated a comprehensive framework outlining the core components integral to TPE interventions (Correia et al., 2022a, 2022b). It was done in liaison with a committee of subject matter experts; post an extensive literature review on patient education and behaviour change techniques, and theories in the field of TPE (Correia et al., 2019; Correia et al., 2022a, 2022b).

We categorized the intervention components into five principal domains: comprehension of disease mechanisms, strategies for disease self-management, adoption of healthier lifestyles, enhancement of cognitive and behavioural resilience, and cultivation of effective interpersonal competencies. This framework, detailed across 22 distinct intervention components, served as a data extraction framework for understanding the multifaceted nature of TPE interventions. This refined taxonomy not only encapsulates the essence of TPE interventions but also guides practitioners in crafting and implementing tailored educational strategies aimed at enhancing patient autonomy and well-being in the context of chronic conditions. The taxonomy of components of TPE interventions along with their definitions is provided in Table 2.

**Table 2.** Taxonomy for therapeutic patient education content in psychiatric disorders

Category	Specific Skill	Definition
Understanding disease processes	Aetiology of the disorder	Understanding the origin or cause of the disorder.
	Behaviours influencing health and illness	Recognizing how specific behaviours can positively or negatively impact the progression and management of the disorder.
	Treatment modalities and options	Familiarity with available treatment options and how they function.
Disease management	Strategies for disease management	Techniques and practices tailored to manage the symptoms and progression of the disorder.
	Techniques for self–monitoring	Tools or practices that help patients monitor their own symptoms and well–being.
	Adapting drug doses and self–initiation of treatments	Knowledge on adjusting medication doses and when and how to initiate specific treatments independently.
	Performance of technical gestures and manoeuvres	Mastery of certain techniques or manoeuvres essential for disease management, like specific exercises or practices.
	Approaches to counteract challenges posed by the disorder	Techniques or strategies that patients can employ to deal with specific challenges or complications arising from the disorder.
Lifestyle changes	Guidance on implementing pertinent lifestyle changes	Recommendations on lifestyle alterations, like diet or activity levels, that can assist in managing or mitigating symptoms.
	Awareness generation regarding health–related risk factors	Education on potential risks or triggers that can exacerbate the disorder or impact treatment effectiveness.
	Prevention strategies for avoidable complications	Techniques or practices that can help prevent potential complications linked with the disorder.
Cognitive and behavioural coping	Building self–confidence and self–awareness	Techniques that enhance one’s trust in their abilities and understanding of their emotions and responses.
	Stress management techniques	Tools or strategies designed to help patients manage and reduce stress, which can be a trigger or complicating factor for many psychiatric disorders.
	Critical thinking, decision–making, and problem–solving tools	Strategies to enhance cognitive abilities, allowing patients to make informed decisions about their health and navigate challenges effectively.
	Goal setting and attainment strategies	Techniques that guide patients in setting realistic and achievable goals related to their disorder management, and methods to reach them.
	Strengthening individual resilience and self–care	Practices and mindsets that bolster one’s ability to recover from setbacks and maintain self–care routines.
	Various coping mechanisms to combat the disorder	A range of techniques that patients can employ to cope with symptoms, emotional challenges, or other aspects of their disorder.
Interpersonal skills	Dissemination of organizational information	Providing patients with necessary organizational details, like how to manage appointments, therapy sessions, or medication schedules.
	Training on effective communication skills	Techniques that enhance a patient’s ability to communicate their needs, symptoms, or concerns with healthcare providers, caregivers, or loved ones.
	Harnessing and building social support networks	Strategies to help patients seek out, build, and maintain supportive relationships and networks that can assist in their disease management journey.

### Data extraction for dissemination methods for intervention

We extracted data regarding the diverse dissemination techniques employed across studies to facilitate participant engagement and enhance the effectiveness of the interventions. The chosen methods varied widely, from interactive workshops to digital media applications, reflecting the evolving landscape of educational and therapeutic strategies. For a comprehensive definition of the dissemination techniques identified in our review, refer to Supplementary Table S1.

### Risk of bias assessment

Risk of bias was evaluated using the Cochrane tool for RCTs, assessing domains such as random sequence generation, allocation concealment, blinding of outcome assessors (Higgins et al., 2019).

### Data synthesis

To summarize findings across included trials, categorical data are provided as frequencies (%) and continuous variables as mean (SD). We used a narrative synthesis approach for analysing content of interventions. We could not conduct a meta-analysis due to heterogeneity in intervention curriculum and measurement of outcomes.

## Results

### Database searching

**Figure 1.**
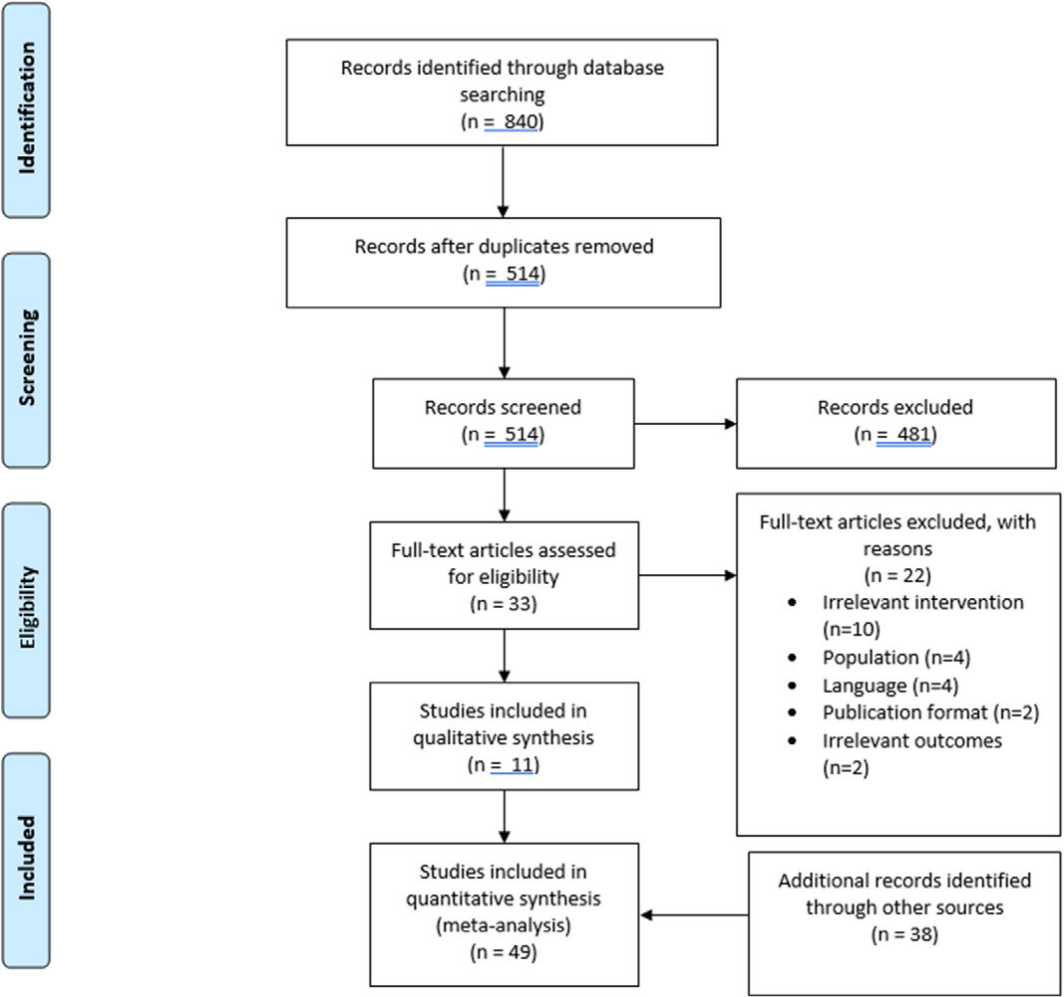
PRISMA flowchart demonstrating the process for selection of studies.

Our systematic database search initially yielded 840 reference records (Figure 1). After the automatic deduplication process, 514 unique records remained for screening. A rigorous evaluation narrowed this down further, leaving 33 records suitable for full-text review. Following a detailed assessment, only 11 articles were ultimately included in our systematic review. Exclusion reasons were as follows: language other than English and French (*n* = 4), abbreviated forms of publication (*n* = 2), irrelevant outcomes (*n* = 2), unrelated interventions (*n* = 10), and unsuitable study population (*n* = 4). We supplemented this database search by including 38 studies from our previously published meta-analysis evaluating effectiveness of TPE interventions across all medical specialties (Correia et al., 2019).

Among 49 included interventions, 13 were aimed at bipolar disorder, depression (*n* = 12), multiple serious mental illnesses and comorbidities (*n* = 11), schizophrenia and psychoses (*n* = 13). Details of each intervention are provided in Supplementary Table S1.

### Intervention delivery

A diverse array of professionals and approaches have been utilized across educational interventions for depression, bipolar disorder, psychosis, and patients with complex clinical presentations. In the realm of depression, nurses have been significant agents, acting as sole interventionists in some cases (Jonkers et al., 2011; Casañas et al., 2012; Raya-Tena et al., 2021). Additionally, general practitioners have been pivotal in delivering psychoeducation in primary care settings (Gili et al., 2020), and multidisciplinary teams involving combinations of psychologists, nurses, and trained health professionals, have been prevalent (Katon et al., 2001; Haringsma et al., 2005; Vera et al., 2010; Aragonès et al., 2012; Hunkeler et al., 2012; Ludman et al., 2013).

In bipolar disorder interventions, diverse professionals such as clinical psychologists (González Isasi et al., 2014; Husain et al., 2017; Buizza et al., 2019), nurses (Simon et al., 2006; Kavitha et al., 2022), and psychiatrists (Bauer et al., 2009; Van Dijk et al., 2013; Luciano et al., 2022) have been instrumental. Additionally, unique agents such as web-based platforms (Ershad Sarabi et al., 2021) and senior psychology students (Cardoso et al., 2015) were also employed.

Regarding psychosis, delivery was predominantly undertaken by allied health professionals such as specialist nurses (Aho-Mustonen et al., 2010; Chien et al., 2016; Chien et al., 2019), researchers (Sato et al., 2012; Alhadidi et al., 2023), and pharmacists (Mishra et al., 2017). Furthermore, interventions were also carried out by social workers (Shin and Lukens, 2002) and psychotherapists (Bäuml et al., 2007; Valencia et al., 2007).

For patients with complex clinical presentations, multidisciplinary teams again played a crucial role. These teams included psychiatrists, psychologists, social workers and physiotherapists (Lara-Cabrera et al., 2016a). Other professionals such as physicians (Aragonès et al., 2019), clinicians (Jensen et al., 2019), nurse practitioners (Druss et al., 2017) and dietitians (Erickson et al., 2017) also contributed, showing the breadth and depth of involvement across various specializations in managing complex cases. Peer-led approaches also demonstrated significant involvement in interventions, emphasizing the value of lived experience in educational strategies (Steigman et al., 2014; Ell et al., 2017; Muralidharan et al., 2019)

### Format of interventions

A total of 21 interventions were delivered in groups followed by individual (*n* = 12), mixed format (*n* = 14) and electronically (*n* = 2). Among interventions delivered to groups, Cechnicki and Bielańska (2017) ran outpatient group interventions with stays in therapeutic hostels and camps. Among interventions delivered to individuals, house visits were conducted in two studies (Chien et al., 2016; Ell et al., 2017). The 12 interventions utilizing a variety of methods, a major proportion delivered a combination of group and individual counselling. Gili et al. (2020) ran face-to-face group sessions and four web-based, individual, and interactive therapeutic modules. Eight interventions utilized either group or individual psychoeducation sessions with telephone-based monitoring (Katon et al., 2001; Simon et al., 2006; Chew-Graham et al., 2007; Ludman et al., 2007; Vera et al., 2010; Aragonès et al., 2012; Ludman et al., 2013; Coventry et al., 2015). Interventions delivered solely electronically used mobile app to deliver psychoeducation in patients with bipolar disorder (Ershad Sarabi et al., 2021) and Internet-delivered care management and patient self-management program, among patients treated for recurrent or chronic depression (Hunkeler et al., 2012). Interventions ranged from a minimum of six sessions (Cardoso et al., 2015; Chien et al., 2016) to a total of 48 sessions (Simon et al., 2006).

### Modes of dissemination and skills targeted by interventions

**Figure 2.**
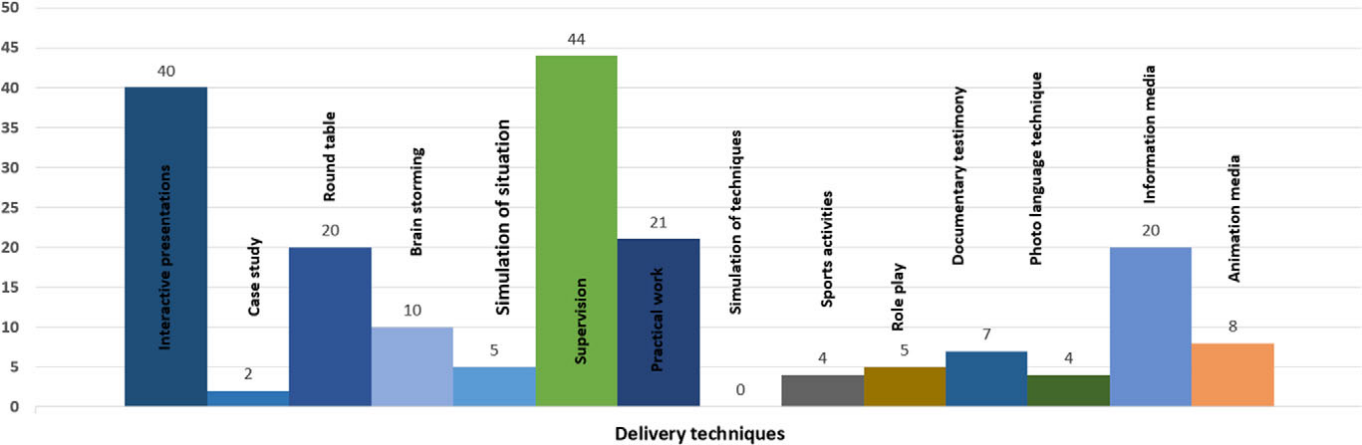
Strategies used for delivery of interventions.

Various methods have been employed for the dissemination of interventions, with a particular emphasis on strategies such as interactive presentations rather than didactic (*n* = 40), round table discussions (*n* = 20), practical workshops (*n* = 21), and the use of information media (*n* = 20). A range of dissemination methods for interventions were used, focusing notably on interactive approaches. These include interactive presentations (*n* = 40), roundtable discussions (*n* = 20), practical workshops (*n* = 21), and the utilization of various information media (*n* = 20), demonstrating a preference for engaging and participatory techniques over traditional didactic methods. Intervention recipients received supervision from delivery agents in 44 studies. Conversely, other strategies like case studies, brainstorming sessions, simulations, sports activities, role plays, documentary testimonies, photo-language techniques and animation media were less frequently utilized (Figure 2).

Examining the different skills imparted during the interventions, the focus predominantly leaned towards the teaching of coping strategies. These encompassed both cognitive and behavioural coping skills, including areas such as self-confidence (*n* = 37), stress management (*n* = 39), critical thinking (*n* = 26), problem-solving (*n* = 18), goal setting (*n* = 31), situational awareness (*n* = 36) and self-care (*n* = 36), with unspecified coping skills also noted (*n* = 32).

**Figure 3.**
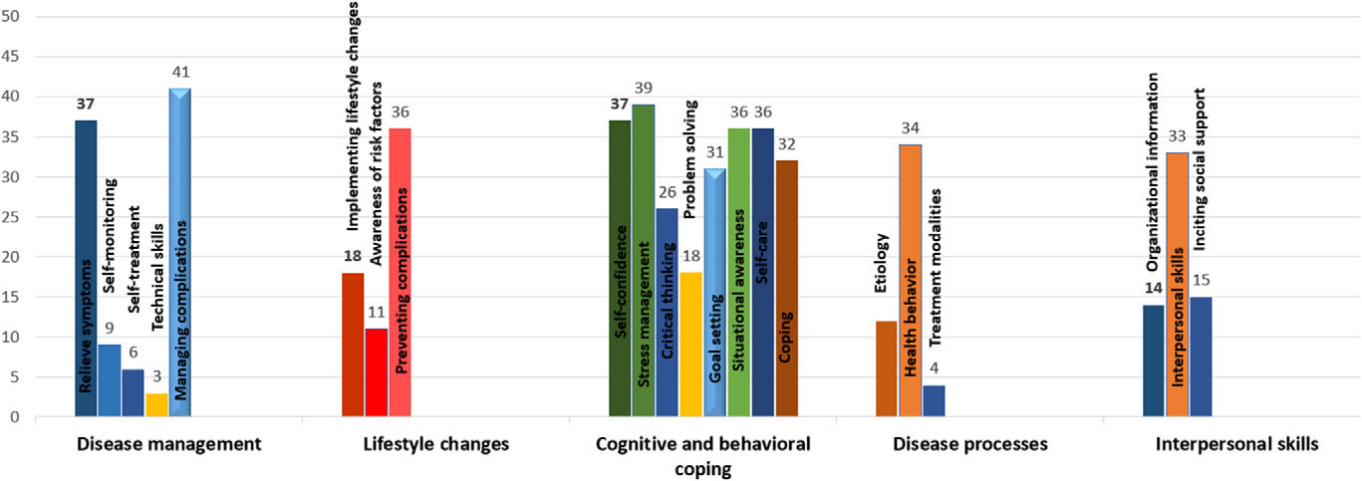
Components of interventions used in TPE interventions.

In the context of disease management techniques, a substantial number of interventions were geared towards teaching symptom relief (*n* = 37) and managing complications (*n* = 41), while aspects such as self-monitoring, initiating self-treatment, and the employment of technical skills were less emphasized. Regarding lifestyle adaptations, the interventions mainly focused on the prevention of complications (*n* = 36) and the implementation of lifestyle changes (*n* = 18). However, there was a lesser focus on building awareness regarding risk factors (*n* = 11). Analysing the disease processes, it was found that health behaviours constituted a significant part of the curriculum in 34 interventions, while there was lesser emphasis on discussing aetiology and treatment modalities. In relation to interpersonal processes, a strong emphasis was placed on developing interpersonal skills (*n* = 33), identifying social support sources (*n* = 15), and acquiring organizational information (*n* = 14) (Figure 3).

### Outcomes in TPE interventions

Interventions aimed at patients with depression were aimed at heterogeneous outcomes and employed varying strategies. Three of these interventions focused on managing depression in primary care (Chew-Graham et al., 2007; Aragonès et al., 2019; Gili et al., 2020), improve therapeutic response and decrease remission rates (Casañas et al., 2012; Raya-Tena et al., 2021), improve quality of life (Casañas et al., 2012); improving coping skills (Haringsma et al., 2005); improve daily functioning and social skills among patients with chronic depression (Vera et al., 2010; Jonkers et al., 2011); self-efficacy in management (Ludman et al., 2013), improving adherence (Katon et al., 2001); reducing recurrence or chronicity by self-management (Ludman et al., 2007; Hunkeler et al., 2012).

For bipolar disorders, outcomes included improvements in early detection (Buizza et al., 2019); acceptability (Husain et al., 2017), functional improvement (Simon et al., 2006; Kavitha et al., 2022; Luciano et al., 2022), reduced recurrence (Colom et al., 2009; González Isasi et al., 2014; Petzold et al., 2019), testing electronic methods of psychoeducation (Ershad Sarabi et al., 2021), concordance with treatment guidelines (Bauer et al., 2009) and reduced hospitalization (Colom et al., 2009). The psychoeducational interventions for schizophrenia were largely aimed at improving longer term therapeutic outcomes, especially reduction of relapse and rehospitalization rates (Hornung et al., 1999; Bäuml et al., 2007; Valencia et al., 2007; Sato et al., 2012; Cechnicki and Bielańska, 2017), medication adherence (Sousa et al., 2013; Chien et al., 2016; Mishra et al., 2017), and improvement in psychosocial functioning and quality of life (Sousa et al., 2013; Chien et al., 2016; Mishra et al., 2017; Chien et al., 2019; Mueser et al., 2021), and decreased internalized stigma (Alhadidi et al., 2023; Shin and Lukens, 2002). One of the interventions was aimed at offenders with schizophrenia in forensic settings (Aho-Mustonen et al., 2010).

For more complex presentations and interventions, aims included to improve patient participation in treatment (Lara-Cabrera et al., 2016a), improved management (Lara-Cabrera et al., 2016a), improved self-management (Steigman et al., 2014; Coventry et al., 2015; Muralidharan et al., 2019), and better collaborative care (Aragonès et al., 2019), improved physical health (Erickson et al., 2017) and improved management of medical and psychiatric illnesses (Bartels et al., 2014).

### Efficacy and risk of bias

A total of 12 interventions (24.48%) reported no statistically significant benefits (Simon et al., 2006; Chew-Graham et al., 2007; Ludman et al., 2007; Jonkers et al., 2011; Hunkeler et al., 2012; Bartels et al., 2014; González Isasi et al., 2014; Ell et al., 2017; Jensen et al., 2019; Petzold et al., 2019; Ershad Sarabi et al., 2021; Mueser et al., 2021). Several studies were noted to be at high risk of bias. Random sequence generation was at low risk in 27 studies, allocation concealment in 11 studies, blinding (*n* = 18), attrition bias (*n* = 21), selective reporting (*n* = 47) and other sources of bias (*n* = 6).

### TPE interventions for depression

*Group-based psychoeducation:* Raya-Tena et al., a psychoeducational group intervention for patients with depression and physical comorbidity, comprising 12 weekly sessions lasting 90 min each (Raya-Tena et al., 2021). Casanas et al. employed a similar approach, offering health education about depression, diet, sleep, exercise, and treatment combined with breathing techniques, problem-solving and behavioural activation (Casañas et al., 2012).

*Web-based and technological methods:* Gili et al. introduced a low-intensity, internet-based intervention for depression, consisting of one face-to-face group session and four individual interactive online modules (Gili et al., 2020). This multi-modal approach was designed to enhance engagement with the intervention. Hunkeler et al. offered a comprehensive internet-delivered program, “eCare for Moods”, accessible 24/7 for a year, including personalized self-monitoring, secure messaging, and monitored discussion groups. There was an emphasis on patient-centric design tailored to individual needs (Hunkeler et al., 2012).

*Telephone-based interventions:* Ludman et al. presented a pilot study offering telephone care management for chronic depression (Ludman et al., 2007). This approach was further complemented by either peer-led or professionally led group programs. In another intervention, patients with chronic illnesses and depression received regular scheduled visits and follow-up phone calls over 12 months, focusing on medication adherence and self-care (Ludman et al., 2013).

*Interventions specifically targeted the elderly population:* Two studies tailored interventions to the unique needs of older people. Haringsma et al. introduced the “Coping With Depression (CWD)” course for older adults, emphasizing skills such as relaxation and social skills over 10 weekly sessions (Haringsma et al., 2005). Jonkers et al. focused on the self-management beliefs and behaviours of chronically ill elderly individuals suffering from depression, guiding them to alter behaviour and draft action plans (Jonkers et al., 2011).

*Specific patient populations:* Vera et al.’s collaborative care model targeted patients with depression and chronic illnesses, offering them therapeutic patient education about depression combined with either cognitive-behavioural therapy or medication (Vera et al., 2010). The INDI project, aimed to improve depression management in primary care, providing psychological and educational support over monthly visits until depression remission (Aragonès et al., 2012).

*Relapse prevention:* Specialized strategies for relapse prevention included long-term adherence to antidepressants, self-monitoring strategies and proactive steps, supported by two in-person visits and multiple phone calls and mailings (Katon et al., 2001).

### TPE interventions for bipolar disorder

*Group interventions:* The Colom and Vieta’s Model focused on a comprehensive range of outcomes (Colom et al., 2009; Husain et al., 2017; Buizza et al., 2019). Specifically, it aims to increase patients’ illness awareness, which involves understanding the nature of their condition and its implications. The model also targets treatment adherence, ensuring that individuals stick to prescribed regimens to optimize therapeutic benefits. Another crucial outcome is early warning signs detection, which trains patients to recognize impending episodes or relapses, allowing timely intervention (Colom et al., 2009). Additionally, the model seeks to establish lifestyle consistency, helping patients maintain a routine that supports mental health stability (Colom et al., 2009). Regarding the providers of these interventions, trained therapists or mental health professionals typically deliver the sessions. The dose of intervention can vary, but it usually consists of regular group sessions spread over weeks or months, offering an environment for shared learning and support.

Some researchers experimented by adapting pre-existing models to cater to their specific research objectives. A case in point is Luciano et al.’s intervention, who modified the Falloon model, originally developed for schizophrenia, to suit the nuances of bipolar disorder (Luciano et al., 2022). This adaptation involved the inclusion of components more relevant to bipolar patients, ensuring more tailored support. On a similar note, Gonzalez-Isasi et al. ventured into an integrated approach. Their model amalgamated aspects like anxiety control, which helps individuals manage their anxiety symptoms better; cognitive restructuring, a technique that aids in changing detrimental thought patterns; and problem-solving training, empowering patients to tackle challenges effectively (González Isasi et al., 2014).

Among these Group Psychoeducation Models, Kessing et al. uniquely underscored the goal of diminishing psychiatric hospital readmissions (Kessing et al., 2013). To achieve this, they amalgamated pharmacological treatments, which provided the necessary medical intervention, with group psychoeducation sessions, facilitating a holistic healing process. In a similar vein, Cardoso et al. delved into psychoeducation’s impact on biological rhythms. Their intervention focused on understanding how regulating biological rhythms could potentially reduce mood symptoms (Cardoso et al., 2015). Over a span of a year, they observed and assessed the link between psychoeducation and its influence on stabilizing these rhythms, leading to a consequent reduction in mood-related symptoms.

*Family-focused interventions:* The principle that the family plays an indispensable role in the therapeutic journey is underscored by family-focused interventions. Such interventions recognize that an individual’s environment, especially immediate family, can play a pivotal role in treatment efficacy. Kavitha et al. championed a novel nurse-led, family-centric approach (Kavitha et al., 2022). The primary outcome this intervention aimed for was the enhancement of bipolar patients’ overall functionality. This meant ensuring that patients were not only symptomatically stable but also capable of fulfilling daily roles and responsibilities effectively. The intervention sessions were designed to be holistic, starting from the basics by introducing the concept of Bipolar Affective Disorder (BPAD) to families. This introductory phase was essential to ensure that families understood the nature of the condition. Post this, the intervention delved into comprehensive management techniques. Families were educated about symptom identification, crisis management, and supportive techniques to ensure adherence to treatment regimens. The intervention was uniquely positioned by being led by nurses, who, with their hands-on experience in patient care, brought in practical insights and strategies to the sessions. The dose of this intervention likely consisted of multiple sessions spread over weeks or months, ensuring a progressive and sustained learning experience for families (Kavitha et al., 2022).

On the other hand, Luciano et al., while also emphasizing family intervention, took a slightly different route (Luciano et al., 2022). The basis of their study was the adapted Falloon model, which originally catered to schizophrenia but was adjusted for relevance to bipolar disorder. The outcomes targeted in this study could have been multi-faceted, involving both symptom management and improvement in interpersonal family dynamics. Given the foundation in the Falloon model, the intervention possibly included components of psychoeducation, skill-building, and crisis intervention tailored for family settings. This approach might have been administered by trained therapists or mental health professionals experienced in the Falloon model’s intricacies. The dose and detailed content would have been structured according to the model’s principles, ensuring a comprehensive understanding and application for the families involved.

A more tailored approach is evident in Individual Psychoeducation Sessions. Both Kavitha et al. and Husain et al. provide interventions on an individual basis, while Simon et al. put forth a structured psychoeducational program, emphasizing feedback to mental health providers and consistent follow-up (Husain et al., 2017; Kavitha et al., 2022; Luciano et al., 2022).

Delving into Special Techniques and Therapies, VanDijk et al. explored dialectical behaviour therapy skills within a group setting, targeting emotional regulation enhancement (Van Dijk et al., 2013). Addressing cultural nuances, special cases like Husain et al. and Luciano et al. (2020) presented a program tailored for Pakistan and Italy, respectively, aligning with its societal and cultural dynamics (Husain et al., 2017; Luciano et al., 2022).

*Collaborative care model:* Bauer et al. (2009) assessed the collaborative care model’s efficiency in bipolar disorder care, emphasizing guideline concordance (Bauer et al., 2009). They explored the efficacy of a collaborative care model for the treatment of bipolar disorder. This model was established on three foundational, manual-based components, which align with the core principles of chronic care models tailored for medical illnesses. Patients were equipped with skills to manage their condition through structured group psychoeducation sessions. This aimed at giving patients the tools and knowledge to understand and better navigate their disorder. Healthcare providers were furnished with evidence-based VA clinical practice guidelines in a simplified, digestible format. This was to ensure that the treatment methods were standardized and based on the latest scientific evidence. The model promoted seamless access to treatment by introducing a nurse care manager who collaborated closely with a designated psychiatrist. This ensured that patients received consistent, uninterrupted care.

*Technological Aids:* Petzold et al. creatively merged the traditional group psychoeducation approach with a contemporary touch (Petzold et al., 2019). Participants not only attended structured, weekly sessions that followed a comprehensive curriculum on bipolar disorder but also engaged in daily electronic self-monitoring. This daily monitoring was facilitated through a user-friendly platform where patients could log their moods, symptoms, and triggers. Such consistent tracking provided real-time insights into their condition, enabling timely interventions and personalized feedback during group sessions. In a parallel tech-forward approach, Sarabi et al. ventured into the world of mobile applications to enhance psychoeducational delivery. Their application, named “BipolarEd”, comprised a series of interactive modules spanning topics from the basics of bipolar disorder, its management, coping strategies, to real-life testimonials (Ershad Sarabi et al., 2021). The application also featured push notifications, reminding users of medication schedules and prompting them to engage in daily mood tracking. By offering a blend of textual, visual, and interactive content, the application aimed to ensure sustained user engagement and enhanced understanding.

Utilizing psychotherapeutic approaches, VanDijk et al. delved deep into the potential of dialectical behaviour therapy (DBT) skills. Implemented in a psychoeducational group setting, the primary aim was emotional regulation enhancement for individuals with bipolar disorder (Van Dijk et al., 2013). The structured program spanned over 12 sessions, each lasting 90 min. The curriculum was segmented into four core modules: mindfulness, distress tolerance, emotion regulation, and interpersonal effectiveness. Through a combination of role-playing, group discussions and homework assignments, participants were equipped with tools to better manage their emotional responses and enhance interpersonal relationships.

### TPE interventions for psychosis

*Content and mode of delivery:* Many studies emphasized the importance of psychoeducation for both patients with schizophrenia and their caregivers. Similarly, Cechnicki et al. implemented a Community Treatment Program where patients and their families received psychoeducation after their first psychiatric hospital admission (Cechnicki and Bielańska, 2017). Aho-Mustonen et al.’s psychoeducation intervention was grounded on the Finnish Schizophrenia Practice Guideline, emphasizing empowerment through knowledge of the illness and hope-promoting strategies (Aho-Mustonen et al., 2010). The Munich Psychosis Information Project study provided booklets and sessions to patients and their key relatives, emphasizing empowerment and coping strategies (Bäuml et al., 2007).

*Psychoeducation-based Interventions undergoing cross-cultural adaptation:* Al-Hadidi et al. and Shin et al. meticulously crafted psychoeducation interventions grounded in scholarly literature. To ensure cultural relevance for persons with schizophrenia in Jordan (Alhadidi et al., 2023) and Korea (Shin and Lukens, 2002), the content was subject to expert panel evaluations. The interventions were tailored to reflect the actual needs of the participants, which were ascertained from an initial survey. Notably, the sessions were conducted in Arabic and followed a group format. The use of group sessions fostered a sense of community, allowing participants to build a supportive social network that served as a buffer against feelings of isolation. Drawing from the literature, Al-Hadidi et al. recognized the therapeutic potential of such communal settings in addressing loneliness (Shin and Lukens, 2002). Conducted in Arabic, the interventions used a group format to combat loneliness and build support networks. Participants’ psychiatric health was assessed at each session’s start. The program comprised seven sessions, addressing: understanding schizophrenia; its diagnosis and treatment; medication adherence and action; management of medication side effects; recognizing and preventing relapses; confronting stigma; and reflecting on individual experiences throughout the program. This structure aimed not just to educate but to create a supportive environment for participants.

Shin et al. tailored their psychoeducational intervention for Korean Americans, focusing on the specifics of the illness, crisis management, and medications (Shin and Lukens, 2002. In an effort to cater to the specific cultural and therapeutic needs of Korean Americans diagnosed with schizophrenia, a specialized psychoeducational program was developed based on the literature’s recommendation for a biopsychosocial approach. Recognizing the Korean American community’s respect for teaching and their preference for didactic over interactive formats, the intervention was designed to be educational, authoritative, and expert-led. The sessions were conducted in Korean by a Korean-speaking psychiatric social worker, with supportive sessions facilitated by a supervised master’s student. These sessions combined lecturing with interactive Q&A and discussions.

The curriculum covered diverse topics, including illness definitions, medication management, relapse prevention, stigma, and communication skills, among others. A unique feature was the incorporation of traditional Korean beliefs and concepts related to health and well-being, aiming to provide context and bridge the understanding of psychiatric illness from both cultural and medical perspectives. To aid comprehension, visual materials were employed, and all written materials were available in both Korean and English. To foster interaction, especially in initial sessions, the clinician took a proactive role in steering group discussions. As an added incentive for attendance, refreshments were provided, and to augment the main intervention, family members were offered parallel sessions.

*Mindfulness and therapy-based interventions*: Chein et al. integrated Kabat-Zinn’s mindfulness-based stress reduction techniques into a psychoeducation program to offer a mindfulness-based psychoeducation program for early-stage schizophrenia patients (Chien et al., 2019). Sousa et al. (2013) used the “Levels Of Recovery from psychotic disorders Scale” (LORS) to enable dialogues, focusing on educating patients about their symptoms (Sousa et al., 2013). Chien et al. applied a motivational interviewing-based adherence therapy, where participants received education about their illness and its treatment (Chien et al., 2016). Lastly, Hornung et al. (1999) combined psychoeducational training with cognitive psychotherapy and key-person counselling for schizophrenic outpatients and their key-persons.

*Web-based and Telehealth Interventions*: Mueser et al. offered web-based psychoeducation and skills training for caregivers of schizophrenia patients (Mueser et al., 2021). Caregivers participating in the study were offered up to 16 sessions of MyHealios, a telehealth-based family psychoeducation (FP) and skills training program spanning 6 months. Each caregiver was paired with a certified masters-level clinician, distinct from the patient’s usual care team. The program’s sessions were conducted via live web-based meetings, lasting 40 minutes each, at times convenient for the caregiver. These sessions encompassed live video feeds of both the caregiver and clinician, with an additional chat window for enhanced interaction.

The program’s content was flexible, allowing caregivers and clinicians to collaboratively choose the topics covered, ensuring information and skills taught were tailored to individual caregiver needs. During the sessions, caregivers discussed the challenges they faced, offering detailed examples, and clinicians provided guidance on handling these challenges. Initially, the sessions were held weekly, with the frequency decreasing over the 6-month period, guiding participants to apply the learned skills in their everyday lives. All caregivers were expected to complete three fundamental modules: engagement and goal setting, communications, and problem-solving and goal achievement. Subsequently, caregivers had the liberty to choose from a range of other modules, such as coping, relapse prevention, and understanding schizophrenia, among others. The table provided gives a breakdown of module completion rates among different groups of caregivers.

*Skills training and psychosocial programs*: Several studies focussed on skills training and psychosocial programs. Valencia et al. implemented a psychosocial skills training approach for out-patients, emphasizing problem-solving to improve communication skills and medication compliance (Valencia et al., 2007). Similarly, Sato et al. rolled out a discharge preparation program for long-term hospitalized patients, utilizing videos and workbooks to provide psychological education and social skills training (Sato et al., 2012).

*Pharmacist-led and medication-focused interventions*: Mishra et al. employed a pharmacist-led approach, providing patients with a medication review followed by an education session. This approach emphasized the importance of medication adherence and quality of life (Mishra et al., 2017).

## Patients with complex clinical presentations

Several studies aimed to bolster patient knowledge, activation, and self-management (Ell et al., 2017; Lara-Cabrera et al., 2016a; Muralidharan et al., 2019; Steigman et al., 2014). Lara-Cabrera et al. educated patients with mental illnesses using PowerPoint presentations and leaflets, promoting an active role in their treatment, awareness of treatment options, and the significance of participation (Lara-Cabrera et al., 2016a). Muralidharan et al. offered education on healthy eating, physical activity, and symptom management, in addition to skills training like goal setting (Muralidharan et al., 2019). Steigman et al.’s BRIDGES intervention empowered participants by enhancing self-esteem and promoting successful mental illness management (Steigman et al., 2014).

In the realm of integrated care for comorbid conditions, several interventions have stood out for their innovative approaches. Erickson et al. designed the “Lifestyle Balance” intervention specifically for veterans grappling with weight gain (Erickson et al., 2017). This intervention was comprised of behaviourally driven techniques, including specialized classes and individual nutritional counselling, aiming to achieve outcomes like weight reduction and improved behavioural choices. On the other hand, Bartels et al. pioneered the I-IMR program, targeting older adults contending with both psychiatric and general medical illnesses (Bartels et al., 2014). The program provided a comprehensive mix of psychoeducation about the illnesses and their treatments, medication adherence training, relapse prevention, social skills training, and counselling about self-management and lifestyle adjustments. The intended outcome was to holistically address both types of illnesses, thereby enhancing the overall quality of life for the participants. Similarly, Druss et al.’s approach emphasized an integrated behavioural health strategy, catering specifically to patients with severe mental illnesses and cardiovascular risk factors (Druss et al., 2017). The content was rich, offering education about lifestyle factors such as smoking and diet and ensuring logistical support for medical appointments, with the overarching goal of improving the quality of care.

Aragones et al. introduced the DROP program, primarily crafted for those facing the twin challenges of major depression and chronic pain (Aragonès et al., 2019). Participants were enriched with a manual of DROP and imparted with psychoeducation on pain comprehension, depression management, and relapse planning. The objective was to equip patients with strategies to manage both conditions effectively. In a more targeted approach, Ell et al.’s “A-Helping-Hand” was formulated for Latinos with comorbid conditions like diabetes and heart disease (Ell et al., 2017). This intervention was unique in its emphasis on depression and self-care management, marrying rapport building, problem formulation, and action planning to achieve improved self-care outcomes. Coventry et al, meanwhile, ventured into an integrated collaborative care model, catering to patients diagnosed with depression alongside diabetes or cardiovascular ailments (Coventry et al., 2015). Their intervention offered brief low-intensity psychological therapy, fostering a deeper understanding of the intricate links between patients’ mood and the management of their physical conditions, thus aspiring to enhance both mental and physical well-being.

## Discussion

### Summary of findings

This systematic review explores TPE interventions to support care for people with severe mental disorders. A diverse range of professionals engaged in delivery of these interventions effectively, leading to improved symptomatology and quality of life among patients receiving these interventions. A higher proportion of interventions were delivered in groups, however, more recently use of electronic media such as mobile apps and telemonitoring devices have been utilized. These interventions were largely grounded in psychotherapy literature. We found that most of the interventions were developed and tested in Western populations.

### Interpretation of findings

The findings from our comprehensive review illuminate the prevalent strategies and skills imparted in TPE interventions for severe mental disorders. A discernible trend in favour of interactive presentations, supervision sessions, and round table discussions was observed. This is indicative of the prevailing preferences, perceived efficacy, and accessibility of these methods within mental health educational settings. These strategies tend to facilitate a more engaging, direct, and personalized educational interaction, essential in the context of severe mental disorders where individualized attention and adaptation of information are crucial. The less frequently utilized methods such as simulations, sports activities, and role plays, despite being dynamic and potentially impactful, might be underutilized due to logistical constraints, lack of training and expertise, or perceived applicability in the context of severe mental disorders (Zhao et al., 2015).

Coping strategies emerged as a pivotal focus of the interventions. While there was a significant focus on preventing complications and implementing lifestyle changes, there seemed to be a lesser emphasis on creating awareness about risk factors and discussing aetiology and treatment modalities. The interventions predominantly emphasized symptom relief and managing complications. This focus reflects a practical approach aimed at equipping individuals with the essential tools to manage the immediate and tangible aspects of their disorders, thereby enhancing their quality of life (Correia et al., 2022a, 2022b). Interpersonal skills were given significant emphasis, recognizing the vital role of social support and interpersonal efficacy in enhancing the resilience and adaptability of individuals with severe mental disorders.

The focus on enhancing cognitive and behavioural coping skills, like self-confidence, stress management, and situational awareness, highlights the crucial role of equipping individuals with severe mental disorders with practical and empowering strategies, enabling them to effectively navigate daily challenges and manage their conditions adeptly (Michie et al., 2013). The pronounced emphasis on coping strategies aligns seamlessly with foundational psychotherapeutic techniques, including cognitive behavioural therapy (CBT), dialectical behaviour therapy (DBT), and mindfulness-based therapies (Butler et al., 2006; Fjorback et al., 2011; Panos et al., 2014). Psychiatric staff, traditionally trained in these psychotherapeutic models, naturally gravitate towards these familiar and well-established techniques, leveraging their expertise to enhance the intervention’s efficacy and relevance. However, these tools represent just a part of the broader and more complex process of therapeutic patient education, which encompasses a comprehensive approach that goes beyond the aforementioned strategies.

This approach has several benefits. Familiarity with these techniques could foster a sense of competence and comfort, enabling practitioners to deliver the intervention with enhanced confidence and proficiency. Utilizing coping strategies that dovetail with psychotherapeutic principles also facilitates a harmonized integration of therapeutic patient education within the broader treatment paradigm. This alignment likely fosters continuity, cohesion, and synergy across various therapeutic touchpoints, promoting a more holistic and integrated approach to managing severe mental disorders. While the alignment with psychotherapeutic principles underscores a strategic congruence, it also invites reflection on the scope of diversity and innovation within therapeutic patient education interventions (Michie et al., 2013). The prevailing focus on coping strategies, potentially influenced by the psychiatric staff’s psychotherapeutic orientation, may inadvertently overshadow or limit the exploration of alternative or complementary strategies.

Despite the evident promise of TPE, its real-world implementation faces multifaceted challenges. From the patient’s perspective, barriers such as stigma, inadequate resources, or lack of access to trained professionals can limit its effectiveness (WHO). In contrast to the recent WHO guide on therapeutic patient education, healthcare providers often lack training in patient education techniques, and these strategies are not widely implemented in clinical settings. (WHO). Practitioners grapple with constraints like limited training, time restrictions, or resource limitations. Globally, mental health challenges manifest diversely across regions and cultures. As such, it becomes paramount to distinguish global patterns from local nuances (Husain et al., 2017; Shin and Lukens, 2002).

The under-representation of low and middle-income countries (LMICs) in the tested interventions also highlights a significant gap in culturally diverse and contextually relevant therapeutic patient education for severe mental disorders. The interventions tested in Jordan, Korea, and Pakistan, although rooted in Western curricula, underscored a crucial pivot towards cross-cultural adaptation to ensure relevance, accessibility, and efficacy within these distinct cultural contexts (Alhadidi et al., 2023; Husain et al., 2017; Shin and Lukens 2002). These serve as important case studies for cross-cultural adaptation of interventions before implementing in LMIC. For instance, Alhadidi et al. (2023) emphasized linguistic and format adaptations, utilizing native languages and group formats to foster accessibility and communal support.

Tailoring content to resonate with the participants’ lived experiences and contextual realities, as seen in the adaptations in Jordan and Korea, underscores the importance of contextually grounded relevance (Alhadidi et al., 2023; Shin and Lukens, 2002). Expert panel evaluations and initial surveys are instrumental in shaping the content to be more attuned to the actual needs and preferences of the participants (Shin and Lukens, 2002). The interventions should demonstrate a sensitivity to cultural preferences, such as the respect for teaching and authoritative delivery seen in the Korean adaptation (Shin and Lukens, 2002). Recognizing and respecting such cultural values and preferences is crucial in enhancing the intervention’s acceptance and resonance. Another pivotal pillar for cross-cultural adaptation underscores recognition of cultural values, such as community and familial ties, was pivotal in tailoring the interventions (Luciano et al., 2022). Utilizing communal settings, focusing on family interventions, and emphasizing supportive networks leverage these cultural assets to enhance the intervention’s impact and sustainability. There is an urgent need to train healthcare providers in therapeutic patient education across various settings, and policymakers should facilitate its widespread implementation for chronic and psychiatric disorders.

### Recommendations for future practice

1. The limited use of these diverse dissemination methods and less emphasis on certain thematic areas may indicate potential areas for enrichment and diversification of TPE strategies. Expanding the repertoire of employed methods and content areas could enhance the comprehensiveness, appeal, and efficacy of therapeutic education interventions, possibly leading to more holistic and sustained benefits for individuals with severe mental disorders. For this, adequate training of health workers in therapeutic patient education is mandatory.
2. The efficacy of different psychotherapies for severe mental disorders is proven. If these psychotherapies are not available for all patients in resource-constrained settings, these strategies become a component of TPE interventions.
3. Development of curricula for training HCPs in TPE could be a low-cost and efficient way to improve outcomes in mental disorders in LMIC.
4. Inadequate resources or lack of access to trained professionals in TPE can limit delivery of TPE interventions in resource-constrained settings. In such cases, intervention delivery models based on peers or digital platforms should be complementary.
5. The interventions must be contextualized to local culture. The reviewed adaptations suggest a diversity of approaches in integrating cross-cultural considerations, ranging from linguistic adaptations to structural and content customizations. Integration of partner patients into the professional HCP teams to co-design and co-produce TPE programs with patient groups should be encouraged to develop any tailored educational programmes.
6. In management of severe mental disorders, a more balanced approach that equally prioritizes long-term preventive strategies through TPE, alongside acute phase management, is crucial for the holistic care of individuals with chronic mental health conditions. Incorporating TPE as a fundamental component of long-term care plans emphasizes the importance of patient empowerment, continuous support, and the cultivation of self-management skills. This shift in focus from acute care to preventative care through TPE can lead to more sustainable health outcomes, reduce the burden on healthcare systems, and most importantly, align treatment strategies with the chronic nature of these disorders.
7. Our review identified several interventions that combine elements from psychoeducation and CBT, primarily targeting therapeutic rather than educational goals. Consequently, these interventions are often designed based on professional standards rather than a collaborative framework with the patients/learners. This can potentially lead to a mismatch between the proposed interventions and the actual needs of the patients. We recommend prioritizing the learning needs of patients and addressing them with proven methods in the field of therapeutic patient education.

### Limitations and strengths

This review has three main limitations. The framework we developed for categorizing the components of TPE interventions represents a step forward in organizing and understanding complex TPE interventions. However, a notable limitation is the need for external validation of this framework to confirm its comprehensive coverage of TPE interventions across a spectrum of psychiatric conditions. Moreover, the current framework does not fully account for the potential overlap among different strategies and components of TPE interventions, which could lead to challenges in distinguishing the unique contributions of each component to the overall effectiveness of the intervention. Addressing this limitation calls for further research, possibly through a Delphi study involving expert consensus, to refine the categorization and ensure it accurately reflects the multifaceted nature of TPE.

Our analysis focused on the components explicitly mentioned in the trial or intervention descriptions, and as such, our findings are limited by the level of detail provided by the original study authors. This limitation potentially led to an underrepresentation of fundamental psychoeducational strategies in our review. Future reviews are encouraged to consider the importance of study setting in evaluating the generalizability and applicability of research findings. Division of interventions according to the intervention setting (inpatient, outpatient and both) can present important insights.

Another limitation is the reliance on a narrative synthesis approach for analysing the content of TPE interventions. While this method is well-suited for exploring the diverse nature of these interventions, it inherently restricts our ability to perform quantitative comparisons of their effectiveness. This limitation is particularly notable given the heterogeneous outcomes and intervention designs across the included studies. A more precise evaluation could be achieved through component network meta-analysis (CNMA), which breaks down complex interventions into individual components to estimate their effects (Tsokani et al., 2023). The CNMA’s additive effects model then reconstructs the overall effect of multicomponent interventions by summing the effects of their components (Tsokani et al., 2023). This approach would allow for a more detailed comparison of TPE components, enhancing our understanding of their individual and combined effectiveness in psychiatric care.

## Supporting information

Waqas et al. supplementary material Waqas et al. supplementary material

## Data Availability

All associated data have been provided as supplementary files.

## Supplementary material

The supplementary material for this article can be found at http://doi.org/10.1017/gmh.2024.68.
