# Supplementary material for: Therapeutic patient education for severe mental disorders: A systematic review
Source: Glob Ment Health (Camb). 2024 Oct 10;11:e78. doi: 10.1017/gmh.2024.68 (PMC11504939; doi:10.1017/gmh.2024.68)
Supplement: Waqas et al. supplementary material [file S2054425124000682sup001.docx]

**Supplementary Table 1: Definition of techniques for dissemination of interventions**

| **Technique** | **Definition** |
| --- | --- |
| **Interactive Presentations** | Engaging presentations that encourage audience participation, questions, and discussions to facilitate active learning. |
| **Round Table Discussions** | A form of academic discussion where participants agree on a specific topic to discuss and debate. Each person is given equal right to participate. |
| **Practical Workshops** | Hands-on sessions where participants actively engage in practical activities related to the subject matter, aimed at skill development or problem-solving. |
| **Information Media** | Use of various media forms (videos, pamphlets, digital content) to provide information, often used to enhance understanding or spread awareness on a topic. |
| **Supervision** | The process where intervention recipients are guided and monitored by delivery agents to ensure the correct application of techniques and principles learned. |
| **Case Studies** | An in-depth investigation of a single person, group, event, or situation over a period of time, used as an educational tool to illustrate a particular point or principle. |
| **Brainstorming Sessions** | Group discussion techniques designed to generate a large number of ideas for the solution to a problem. |
| **Simulations** | Replication of real-world processes or systems in a controlled environment for training, analysis, or prediction. |
| **Sports Activities** | Physical activities engaged in for pleasure, including games and exercises, often used to enhance team-building or physical well-being. |
| **Role Plays** | Dramatic techniques in which individuals adopt and act out roles of specific characters, often used in training to explore particular scenarios or challenges. |
| **Documentary Testimonies** | Use of documentary-style videos or audio recordings featuring personal accounts and experiences related to the subject matter. |
| **Photo-Language Techniques** | A method involving the use of photographs or images to stimulate discussion, convey complex ideas, or explore attitudes and emotions. |
| **Animation Media** | Use of animated content to explain concepts, tell stories, or provide educational information, often used to engage and retain the audience's attention. |

**Supplementary Table 2: Characteristics of included studies (n=49)**

| **Reference** | **Brief name** | **Why?** | **Disorder** | **What? (materials and procedures)** | **Who provided?** | **How?** | **When and How Much ?** | **Improvement** |
| --- | --- | --- | --- | --- | --- | --- | --- | --- |
| Buizza 2019 | psychoeducation on bipolar disorder patient | For improving four main areas: illness awareness, treatment adherence, early detection of warning signs of a probable episode, and lifestyle regularity | Bipolar disorder | 21 weekly sessions Group psychoeducation was performed according to Colom and Vieta's model of 90 min | clinical psychologists | in groups | 21 weekly sessions of 90 min | Longer time free from hospitalizations |
| Hussain 2017 | psychoeducation for bipolar disorder patient | feasibility and acceptability of a culturally adapted bipolar psychoeducation programme | Bipolar disorder | intervention group: this group receive psychoeducation on the bipolar. Control group: control group or TAU group This group of patients received routine treatment, which in Pakistan means attending the outpatient clinic and taking prescribed medication. | psychologist | individual | 12 psychoeducation sessions, one session per week, that were administered on an individual patient basis with 1 hour duration | Better satisfaction with care, medication adherence, knowledge and attitudes, quality of life and lower mania and depression symptoms. |
| kavitha 2022 | functional improvement of patient with bipolar disorder | improvement in functional level of patients with BPAD | Bipolar disorder | intervention group:The experimental group received FFNI in seven sessions, along with routine psychiatric treatment. Control group: The control group received routine psychiatric treatment such as medicine and standard ward routines. | nurse | individual session or a family session | seven session duration of each session 30-45 mins | Functional improvement. |
| luciano 2022 | efficacy of psychoeducation on bipolar disorder | improvement of patients’ global psychopathological status and global functioning and reduction of relatives’ objective and subjective burden and improvement of coping strategies | Bipolar disorder | intervention group: with (TAU) intervention group received The experimental intervention is based on the psychoeducational family intervention. Control group: TAU | psychiatrist | individual | Sessions are provided every 10 days (three times a month) for 4 to 6 months (about 12–18 sessions in total). Each session lasts about 90 min. | Patients' clinical status, global functioning and patients and relatives' objective and subjective burden improved. A significant improvement in the levels of perceived professional support and of coping strategies. Reduced relapses, hospitalizations and suicide attempts. |
| petzold2019 | psychoeducation along with electronic monitoring in bipolar disorder | Reduce recurrence | Bipolar disorder | intervention group: psychoeducational group sessions followed by daily self-monitoring along with TAU control group: TAU | therapist | in groups | The duration of intervention per patient was to be 60 weeks (6 weeks PE or supportive counseling plus 54 weeks computer-based self-charting or diary, respectively). Length of each session is 90 mins | No significant benefit reported |
| Sarabi 2021 | Effectiveness of Mobile-based Psychoeducation in Patients with Bipolar Disorder | effectiveness of mobile applications in patients with bipolar disorder | Bipolar disorder | intervention group: the intervention group after discharge from the hospital were taught how to use the application. Control group: control group did not receive the application and received routine health care services during the study. | no one | individual | for 3 months after discharge from hospital | No significant benefit reported |
| Bauer 2009 | Multicomponent collaborative care model for bipolar disorder. | To assess effects of the model on guideline concordance in care. | Bipolar disorder | At hospital discharge, participants were randomized to collaborative or usual care. Collaborative care included provider support through simplified practice guidelines, patient skills management enhancement through psychoeducation, and facilitated treatment access. | psychiatrist, nurses trained in use of simplified summary guideline based on 1997 VA Clinical Practice Guidelines for Bipolar Disorder. | face to face, individually or in group. | 3-years; assessment at baseline and 6 months. | Significantly higher rates of guideline-concordant antimanic treatment |
| Cardoso 2015 | Psychoeducation intervention on patients with bipolar disorder (BD). | To evaluate effect on biological rhythm and in reduction of depressive, anxious, and manic symptoms at 12 months‚Äô follow-up. | Bipolar disorder | Following psychiatric evaluation, in addition to usual care, intervention patients received psychoeducation emphasizing biological nature of the disorder. Structured action plan offered, importance of pharmacological treatment emphasized. Control group: as usual. | senior psychology students. | face to face. | recruitement: June 2010 to June 2012;  program: 6 weekly sessions, 1h each. Follow-up at 6 and 12-month. | Improved biological rhythm, no improvement in depression, |
| Colom 2009 | Group psychoeducation for people with bipolar disorders. | To prevent recurrences and to reduce time spent ill. | Bipolar disorder | In addition to standard pharmacological treatment, intervention group received psychoeducation, aimed at improving illness awareness, treatment adherence, and early detection of prodromal symptoms. | psychologists experienced with bipolar patients, trained on patients‚Äô group management. | groups of 8-12.. | 6 months: 21 sessions of 90 min each; 5-year follow-up. | Fewer episodes and longer time to recurrence, fewer number of days of hospitalization |
| Gonzalez 2014 | Psychoeducation and cognitive-behavioral therapy for patients with refractory bipolar disorder. | To enhance patients' understanding of their disorder, reduce number of hospitalizations, level of anxiety, and improve social skills. | Bipolar disorder | In addition to their pharmacological treatment, intervention patients received psychoeducation, training in anxiety control techniques, cognitive restructuring and problem-solving.  Control group: pharmacological treatment only. | clinical psychologist assisted by psychiatric nurses. | face to face, groups of 10 subjects. | 20 weekly sessions of 1.5 hours each | No improvement in depression |
| Kessing 2013 | Treatment in specialized out-patient mood disorder clinic in early course of bipolar disorder. | To reduce readmission to psychiatric hospital. | Bipolar disorder | Material: manual for psychoeducation. Procedure: Intervention participants received pharmacological treatment and group psychoeducation; cognitive‚Äìbehavioral therapeutic approaches included.  Control group: standard  psychiatric care. | psychiatrists, psychologists, nurses and a social worker with experience in bipolar disorder. | face to face, in groups of 6-8 patients. | 2 years program: settling-in: 6 months; group sessions of 1.5 h weekly for 12 weeks followed by 3 booster sessions; patients then joined 3‚Äì6 months discharge group. | Reduced rates of readmission, better adherence to medication and better satisfaction |
| Simon 2006 | Multicomponent intervention program in bipolar disorder. | To improve quality of care and long-term outcomes. | Bipolar disorder | Intervention: structured psychoeducational program, monthly phone monitoring of symptoms and medication adherence, feedback to treating mental health providers, facilitation of follow-up, and as-needed outreach.  Control group: usual care. | nurse care managers with at least 5 years of clinical psychiatric experience. | in groups and by phone | 24-month program; 5 weekly group sessions (phase 1),  followed by 2xmonthly sessions (phase 2), for a total of 48 sessions. | No improvement in depression |
| VanDijk 2013 | Dialectical behavior therapy in bipolar disorder (BD). | To reduce depressive symptoms, improve affective control and mindfulness self-efficacy in BD. | Bipolar disorder | Psychoeducation about BD, targeting emotion dysregulation and aiming increase patients' self-control and ability to manage distressing thoughts and emotions; mindfulness techniques. Wait-list control group. | psychiatrist | in groups. | twelve 90-min sessions over 12 weeks. | Improved depression |
| Aragones 2012 | Multi-component program for managing depression. | To improve management of depression in primary care (PC). | Depression | Materials are available at www.projecteindi.cat. Procedures: Intervention group received program based on chronic care model. Patients were provided with psychological and educational support, particularly adherence to treatment, social and family relationships. Control group: usual care. | Trained PC nurses and doctors; training consisted of a one-day workshop once a year for 3 years and a 2-h session every quarter to consolidate and update the knowledge and skills at diagnosing depression, evaluating suicidal risk, and treating and monitoring depression. | one to one | enrollement: June 2007-June 2009; intervention: monthly visits untill remission of depression | Improved depression, physical QoL not improved |
| Casanas 2012 | Psycho-educational program for major depression in primary care (PC). | To reduce depressive symptoms and improve  patients‚Äôwell-being and QoL. | Depression | Following assessment, intervention group was provided: 1-health education about depression, diet, sleep, exercise, treatment; 2- breathing techniques; 3- problem solving, and behavioral activation.  Control group: usual care. | nurses with 40 h training in depression | in groups | December 2008 - April 2010; 12 weekly, 1.5 h sessions; assessments: at baseline, at 3, 6 and 9 months. | Improved depression, physical QoL not improved |
| Chew-Graham 2007 | Primary care Intervention for Depression in Elderly (PRIDE). | To assess effectiveness of this model. | Depression | Intervention group was provided education on depression, medication; a facilitated self-help program as well as sign-posting to other services, particularly voluntary agencies. Control group received usual GP care. | community psychiatric nurse (CPN) based in PC with regular access to advice from an old-age psychiatrist. | face to face, in each patient‚Äôs home or by telephone. | 12 weeks, 6 sessions face to face, 5 sessions by phone. Assessments: baseline, at 4-, 8- and 12-weeks. | No improvement in depression |
| Gili 2020 | internet based intervention for depression |  | Depression | All interventions were composed of one face-to-face group session and 4 web-based, individual, and interactive therapeutic modules. Control group: All the patients included in the study (irrespective of the treatment group randomly assigned) received iTAU. | general practitioner | in groups and individual | The **face-to-face session**, which took place in primary care centers, involved up to 5 patients and was 90 min long, The **web-based therapeutic modules** are oriented to work on different psychological techniques, and the duration of each module is approximately between 40 and 60 min for 4-8 weeks | Improved health-related quality of life, negative affect, and well-being in patients with depression. |
| Haringsma 2006 | Coping With Depression (CWD) course for older adults. | To analyze immediate effectiveness of this program. | Depression | CWD participants were taught skills as relaxation, increasing pleasant activities, constructive thinking, improving social skills, and maintaining treatment gains.  Waiting list controls received CWD after the intervention finished. | health professionals trained in conducting CWD. | in groups of 6 - 13 participants. | 10 weekly sessions of two hours each. | Long term remission in depression |
| Hunkeler 2012 | eCare for Moods: Internet-delivered care management and patient self-management program, on patients treated for recurrent or chronic depression. | To improve outcomes of these patients. | Depression | All participants received usual mental health care. In addition, eCare group received personalized self-monitoring, secure messaging with eCare manager, depression education, monitored discussion group, task lists, and appointment calendar. | clinical nurse specialist and a psychologist supervised eCare managers. | internet | eCare was available 24hours a day, seven days a week, for 12months. | No improvement in depression |
| Jonkers 2012 | Minimal psychological intervention (MPI) on self-management beliefs and behaviors in depressed chronically ill elderly persons. | To examine effects on anxiety, daily functioning and social participation. | Depression | All patients received usual care. In addition, intervention patients were guided to alter their behavior and draw up action plan. | trained research nurses. | home visits. | maximum of 10 visits over a period of no more than 3 months. | No improvement in anxiety, self efficacy |
| Katon 2001 | Relapse prevention in chronic depression. | To improve antidepressant adherence and depressive symptom outcomes. | Depression | Material: book, videotape. Procedure: Intervention participants followed specific self-treatment goals: to improve long-term adherence to antidepressants, increase use of self-monitoring strategies and proactive steps.  Control group: usual care. | depression prevention specialists: psychologist, nurse practitioner with a master‚Äôs degree in psychosocial nursing, social worker; they participated in 2 half-day training sessions with psychiatrist. | individually, face to face or telephone monitoring. | 2 visits (one 90-min initial and one 60-min follow-up sessions); 3 phone calls at 1, 4, and 8th months and 4 personalized mailings at 2, 6, 10, 12th months. | Improved medication adherence |
| Ludman 2007 | Intervention programs to provide effective care for chronic or recurrent depression. | To assess different additional intervention services to these patients. | Depression | In addition to usual behavioral health care, intervention patients were offered either: 1- telephone monitoring and care management, or 2- care management plus peer-led chronic-disease self-management group program, or 3- care management plus professionally led depression psychotherapy group. Control group: usual care only. | 1: trained care manager (master‚Äôs-level counselor), 2: trained peer leader, 3: psychologist, trained in CBT, with 8 years of experience treating depression. | 1: by phone, 2 and 3: in groups. | 1: monthly during the first 3 months, then at intervals according to symptoms, medication adherence, and side effects. 2: 6 week workshop, weekly action planning supplemented with bi-monthly problem solving. 3: 10 consecutive weeks, followed by 6 months of 2x monthly ‚Äúbooster‚Äù sessions. | No statistically significant benefit |
| Ludman 2013 | Collaborative care intervention for chronic illnesses and depression. | To improve confidence of patients in ability to follow through with medical regimens and to maintain lifestyle changes even during times of stress. | Depression | Intervention patients received self-care materials: Depression Helpbook, DVD, blood pressure and blood glucose meters. They were followed to support medication adherence and self-care, problem solving and behavior activation. Control group: usual care. | intervention nurses, physicians. | face to face, individually, using motivational counseling and brief negotiation methods. Follow-up phone calls. | In scheduled visits every 2 to 3 weeks, follow-up telephone calls every 4 to 6 weeks, over 12 months. Assessments: baseline, at 6- and 12-months. | Better confidence in management, self efficacy, and stress management |
| raya-tena 2021 | psychoeducational intervention in patient with depressok and physical co-morbidity. | to decrease the remission rate and increase the theraputic response | Depression | intervention group: psychoeducation control group: Members of the CG receive their usual treatment | nurse | in groups | 12 weekly sessions lasting 90 min with 8-12 participants in the group | Effective for remission at long-term and for therapeutic response at short-term |
| Vera 2010 | Collaborative care model for depression. | To reduce depressive symptoms and improve social functioning of depressed patients with chronic illness. | Depression | Education about depression besides one of two optional initial treatments: cognitive-behavioral therapy (CBT) or antidepressant medication. Control group: usual care. | 1-care: master‚Äôs-level counselors or psychologists, who received 12h of training. 2-CBT: psychologists with master‚Äôs or doctoral degrees, who received 24h of CBT training. | in person or by phone. | every 2 weeks initially, then monthly, for up to 6 months.  13 CBT sessions. | Improved depression |
| Lara-Cabrera 2016 | Brief educational programme added to mental health treatment. | To improve patient activation and participation in treatment. | Complex presentation | Material: PowerPoint presentation, leaflets. Procedures: Before they started their usual treatment, intervention patients participated in educational seminar aiming to encourage them to adopt an active role in their treatment.  Control group: usual care only. | psychiatrist, psychologist, social worker, physiotherapist, trained peer-educators. | discussions in groups of 9-15 patients. | Enrollment: October 2010-May 2012. intervention: four hours. Assessments: at baseline, at 1- and 4-months. | Better self-management |
| Lara-Cabrera 2016 | Peer co-led educational programme delivered before mental health treatment. | To improve participants‚Äô knowledge of treatment and self-management. | Complex presentation | Intervention patients were provided education on treatment options, self-management, and importance of their participation, followed by individual planning session.  Control group: usual treatment. | health professionals and trained peer educators. | in small groups. | June 2009-August 2012. two 4-hour group educational sessions; 45-min individual planning session, within a week after group sessions. Assessments: at baseline, at 1-month following the delivery. | Better patient satisfaction and treatment participation |
| Muralidharan 2019 | Living Well: An intervention for adults with serious mental illness. | To improve medical illness self-management. | Complex presentation | Intervention group received education on 1-healthy eating, physical activity, and medication and symptom management; 2- skills training in goal setting, action planning, and problem solving.  Control group: focus on common challenges. | Group leadership shared equally by trained peer and nonpeer facilitators. | in groups. | 12 sessions, 75min each. Assessments: baseline, posttreatment, and follow-up. | Improved mental quality of life, no significant effect of physcial QoL |
| Aragones 2019 | Collaborative care intervention for managing major DepRessiOn and chronic musculoskeletal Pain (DROP). | To assess effectiveness of DROP program in primary care. | Complex presentation | Material: manual of DROP. Intervention included optimized management of depression, psychoeducation on pain understanding and relapse plan.  Control group: usual care. | physicians ( participated in 90 min informing session),  care manager ( psychologist with expertise in management of chronic pain). | face to face and in groups of max 8 patients; homework after each session, reviewed at the beginning of each following session. Follow-ups by phone. | study: June 2015- December 2017; psychoeducational program (first three months): 9 weekly sessions, 2h each; follow-ups: monthly during the first 3 months and then every 3 months up to one year. | Improved mental quality of life |
| Coventry 2015 | Integrated collaborative care for people with depression comorbid with diabetes or cardiovascular disease. | To reduce depression and improve self management. | Complex presentation | Intervention group received brief low intensity psychological therapy. Link between patients' mood and management of their diabetes and/or heart disease was explored. Control group: usual care. | psychological wellbeing practitioners. | face to face and by phone | up to 8 sessions of 30-40 min over 3 months. | Improvement in depression, self management and satifaction with care |
| Druss 2017 | Integrated behavioral health home. | To improve quality of care for patients with serious mental illness and cardio-vascular risk factors. | Complex presentation | Behavioral health home patients were provided education for lifestyle factors (e.g., smoking, diet) and logistical support to ensure that they were able to attend medical appointments. Control group: standard care. | nurse care manager and part-time nurse practitioner | face to face, individually | weekly, for 12 months. | physcial QoL not improved |
| Ell 2017 | A-Helping-Hand (AHH) intervention: depression and self-care management among predominantly Latinos with diabetes and/or heart disease. | To reduce depression symptoms, barriers to care, and activate self-care management. | Complex presentation | Intervention patients were engaged in rapport building, problem formulation, action planning and evaluation (feedback). | bilingual promotoras that received weekly training, following study manual. | face to face, in patients' home. | 6 weekly sessions followed by 3 monthly booster sessions. Assessments repeated at 6 and 12 months. | No significant benefit reported |
| Erickson 2017 | Behavioral weight management "Lifestyle Balance"(LB) for veterans with mental illness . | To address weight gain by behavioral approach. | Complex presentation | Intervention group received "LB", consisting of classes and individual nutritional counseling (NC). Control group received "Usual Care" consisting of weight monitoring and provision of self-help. | trained registered dietitians (RDs). | LB: group classes, multi-modal techniques including colored handouts, written materials, food models.  NC: individually | Recruitment: Sept 2010 - March 2014. Participants met weekly for the first 8 weeks and monthly through month 12. Assessments: baseline and months 2, 6, and 12. | More weight loss, and improved waist circumference and body fat; better adherence to dietary intervention |
| Steigman 2014 | Building Recovery of Individual Dreams and Goals Through Education and Support (BRIDGES): self-management (SM) intervention for mental illness. | To assess effectiveness of BRIDGES intervention. | Complex presentation | BRIDGES: empowerment focused on self-esteem enhancement and encouragement in capacities of engagement in actions allowing to manage mental illness successfully.  Wait-list control group. | peer-led intervention. | face to face, in groups of 4-13 people. | Classes of 2.5h, once a week for 8 weeks | Improved depression |
| Bartels 2014 | Integrated Illness Management and Recovery (I-IMR) for psychiatric and general medical illness for adults aged 50 or older with serious mental illness. | To evaluate program combining training in self-management for both psychiatric and general medical illness. | Complex presentation | Intervention group: psychoeducation about illness and treatment, medication adherence, training in relapse prevention, social skills training; and counseling on self-management and lifestyle changes for chronic conditions. Control group: usual care. | I-IMR specialist with master‚Äôs degree in social work who received 1.5 days of training in administering I-IMR, primery care nurses. | face to face, individually. | November 2006 - March 2009. I-IMR: 8 months;  10 modules, delivered in weekly sessions; 10- and 14-month follow-ups. | No improvement in anxiety, self efficacy |
| Jensen 2019 | Illness Management and Recovery (IMR) rehabilitation program for people with severe mental illness. | To improve illness self-management and help people achieve clinical and personal recovery. | Complex presentation | All participants received treatment as usual, consisting of individual and group therapy and psychoeducation. In addition, intervention participants learned about mental illness, recovery strategies, building social support, and healthy lifestyle. | clinicians at community mental health center. | in groups of 10 subjects. | 9 months, weekly sessions. | No improvement |
| Chein 2019 | psychoeducation program for early stage schizopherenia | significantly greater improvements in their psychosocial functioning (primary outcome) and illness insight and larger reductions in psychotic symptoms and re-hospitalization rates | Schizophrenia | **treatment group1**: The participants received a six-month MPGP (12 two-hour sessions biweekly; 14e16 subjects/group) that was modified from Kabat-Zinn's MBSR and integrated into a psycho-education programme **treatment group2:** Conventional CPGP was implemented similar to MPGP (12 two hour sessions biweekly; 13e16 subjects/group).  **Control Group:** The patients in TAU received only routine community psychiatric and mental healthcare services, which were similar across the clinics attended by all of the study groups. | psychiatric nurse specialist | in groups | treatment group1: 12 two-hour sessions biweekly; 14e16 subjects/group) for 6 months treatment group2: 12 two hour sessions biweekly; 13-16 subjects/group for 6 months | High improvement in functioning, duration of psychiatric re-hospitalisations, psychotic symptoms, and illness insight |
| Majdi Alhadidi | psychoeducation effects on schizopherenic patient | knowledge about schizophrenia, insight into illness, and internalized stigma in persons | Schizophrenia | Each session lasted approximately 45 to 60 minutes and a variety of educational tools (e.g., PowerPoint® slides, photographs, white boards, group discussions, booklets, simulations) were used to enhance participants’ learning and maintain their attention. | Psychoeducation sessions were conducted by the primary researcher | seven small groups of 8 to 10 participants per group | 45-60 mins and total 7 session weekly basis on every other day | Improved knowledge and insight and reduced stigma |
| mueser 2022 | psychoeducation to the caregivers of schizophernia patient | to educate about schizophrenia and its treatment and skills training to improve communication, problem solving, and coping | Schizophrenia | intervention group: psychoeducation and skills training sessions within a 6-month period control group: usual care consisted of caregiver support that was customarily provided by the study site | psychologist | individual | 16 web based session and each session was 40 minutes in length and was conducted on the web at a time convenient for the caregiver. Sessions were planned to occur weekly at the beginning of the program and decrease in frequency over the next 6 months | No significant benefit reported |
| Aho-Mustonen 2011 | Psychoeducation for long-term offender patients with schizophrenia. | To test effects of a brief psychoeducation program for offenders with schizophrenia resident in a high-security hospital. | Schizophrenia | Material: Finnish Schizophrenia Practice Guideline; manual for the programme. Procedures: Both groups received usual treatment. Intervention group received additional psychoeducation to empower participants with knowledge on illness, stress, and medication; hope-promoting strategies emphasized. | Researchers conducted assessment interviews; sessions were conducted by nursing staff of the hospital. They were 2 group leaders who had undergone a 2-day training. | assessment interviews: face to face. Intervention: in groups of 3-8 patients. | In 2006. Intervention: 8 sessions once a week; 45‚Äì60 minutes each.   3-month follow-up assessment. | Knowledge about illness, self-esteem and insight into the illness |
| Bauml 2007 | Psychoeducation in Schizophrenia: Munich Psychosis Information Project (PIP). | To investigate long-term effects of psychoeducation in regard to rehospitalization rates and hospital days. | Schizophrenia | Material: booklets. Procedures: During their index stay, intervention patients and their key relatives each received separate psychoeducational group therapy: they learned how schizophrenic psychoses are provoked and that patient must be treated consistently so empowerment can be developed. Coping strategies discussed. Control group: usual care. | psychotherapists. | face to face, in groups (depending on whether patient could tolerate a group setting). | intervention patients: 4 weekly sessions of 60 min. each, followed by 4 monthly sessions. Relatives (separately): 8 biweekly sessions of 90 to 120 min each.  7-year follow-up period of both groups. | Reduced use of sedatives, hospitalization and mean number of days spent hospitalized |
| Cechnicki 2017 | Community Treatment Program (CTP) for patients diagnosed with schizophrenia. | To assess long-term treatment outcomes. | Schizophrenia | Following first psychiatric hospital admission, control patients remained under individual care. CTP experimental patients and families were provided psychoeducation and group psychotherapy. In both groups optimal doses of neuroleptics were monitored. | psychologists and nurses; doctors were consulted only about medication. | participation in outpatient groups: stays in therapeutic hostels, camps. | 3 years.  Assessments: after first hospitalization, and then after 3 and 12 years. | Improved functioning, severity of symptoms and rate of relapse. |
| Chien 2016 | Motivational-interviewing-based adherence therapy (AT) for schizophrenia. | To improve level of medication adherence, readmission rate, mental status, and level of functioning. | Schizophrenia | All participants received usual psychiatric care. In addition, intervention group was engaged in addressing concerns in medication adherence; was provided education about illness, treatment and its side-effects; and relapse prevention. | community psychiatric nurse (nurse therapist), who received training and supervised practices. | face to face during home visit. | 12 weeks; six 2-h sessions every 2 weeks. | Improved medication adherence |
| Hornung 1999 | Psychoeducational psychotherapeutic program for schizophrenic outpatients and their key-persons. | To examine long-term effects on  re-hospitalization rates. | Schizophrenia | Participants were randomized to one control group and four treatment groups providing different combinations of following treatment strategies: 1) psychoeducational training for medication management; 2) cognitive psychotherapy; and 3) key-person counselling. | staff members | groups of 6-8 patients. | 10 sessions of psychoeducational training, first 5 weekly and remaining 5 biweekly, followed by 15 sessions of cognitive psychotherapy, 7 weekly and 8 biweekly. 20 sessions of key-person counselling. Assessment: baseline, immediately after completion of treatment and after 1, 2, and 5 years. | Fewer rehospitalizations |
| Mishra 2017 | Pharmacist-led collaborative patient education for schizophrenia patients. | To improve medication adherence and quality of life. | Schizophrenia | In addition to usual psychiatric care, intervention patients received medication review, followed by education session using PILs developed for them.  Control group: usual care only. | trained pharmacist. | individually. | 3 follow-ups; during each, medication adherence and QOL were assessed. | Improved medication adherence, physical QoL |
| Sato 2012 | Discharge Preparation Program (DPP): psychosocial program for patients with schizophrenia. | To prepare long-term hospitalized patients with schizophrenia for discharge from hospital. | Schizophrenia | DPP: psychological education, social skills training. Primary tools: video containing various scenes and behaviors, workbook.  Wait-list control group participated after waiting period of 6 months. | at least two staff members. | in groups of 8-10 . | 24 sessions; 1 or 2 sessions per week; (60-90 min each). 17 indoor, 7 outdoor sessions (Practice Program): participants used knowledge and skills learned indoors and gained clear vision of living in community. | Better disharge support for patients |
| Shin 2002 | Psychoeducational intervention for Korean Americans with diagnosis of schizophrenia. | To improve understanding of mental illness leading to decreased perception of stigma, and greater coping skills. | Schizophrenia | Both groups received individual support. In addition, intervention group received education on illness, crisis and its management, medications and side effects. | Korean-speaking psychiatric social worker. | in groups of 8 participants, and individually. | 10 weekly group sessions and 10 weekly individual sessions of 90 minutes. | reduced symptom severity and perception of stigma and greater coping skills |
| Sousa 2013 | "Levels Of Recovery from psychotic disorders Scale"(LORS) -Enabled Dialogue (LED). | To decrease symptoms, improve adherence to medication and functioning. | Schizophrenia | Education about patients' symptoms, according to LORS discrepancy scores. Specific goals, along with behaviors necessary to achieve them, identified for each patient.  Control group: placebo intervention. | doctorate-level clinicians trained in both LORS assessment, and LED intervention. | face to face, individually. | weekly for inpatients, monthly for outpatients, sessions of 30-45 min, over 4 months. Assessments: baseline, and then monthly. | Decrease in psychopathology, as measured by the PANSS and LORS-clinician scores, and an improvement in functioning |
| Valencia 2007 | Psychosocial skills training (PSST) applied to chronic out-patients with schizophrenia. | To reduce symptoms, prevent relapse and rehospitalization, and improve psychosocial functioning(PSF), global functioning and treatment adherence. | Schizophrenia | PSST oriented to problem solving to improve communication skills, recognition of warning signs of relapse, medication compliance, doctors' appointments. Family therapy (FT). Both groups: usual care. | therapists (trainers) were two psychologists (postgraduates in clinical psychology). | in groups of 8 patients. | PSST: 1 h15 min, once a week for a total of 48 sessions during 1 year. FT: 8 group sessions (patients‚Äô relatives only), and 4 sessions for each family, including patient. | Improved medication adherence |

**Supplementary table 3: Risk of bias in included studies**

| **Reference** | **Random sequence generation** | **Allocation concealment** | **Blinding of outcome assessors** | **Attrition bias** | **Selective reporting** | **Other sources of bias** |
| --- | --- | --- | --- | --- | --- | --- |
| Abazernejad, 2019 | L | U | U | L | L | U |
| Aho-Mustonen, 2011 | L | U | U | U | L | H |
| Alhadidi, 2022 | U | U | U | U | L | H |
| Al-HajMohd 2016 | L | U | U | L | L | H |
| Aragones, 2012 | L | L | L | L | L | L |
| Aragones, 2019 | L | U | L | L | L | L |
| Bauml, 2007 | U | U | U | H | L | H |
| Bartels, 2014 | U | U | U | H | H | H |
| Bauer, 2009 | U | U | U | U | L | H |
| Buizza, 2019 | H | H | H | L | L | H |
| Cardoso, 2015 | U | U | H | H | L | H |
| Casanas, 2012 | L | U | H | L | L | L |
| Cechnicki, 2017 | U | U | U | U | L | H |
| Chew-Graham, 2007 | L | L | U | L | L | H |
| Chien, 2016 | L | L | L | L | L | H |
| Chien, 2019 | L | U | L | U | L | U |
| Colom, 2009 | L | U | L | L | L | L |
| Coventry, 2015 | L | L | H | L | L | H |
| Druss, 2011 | L | U | L | H | L | L |
| Ell, 2017 | L | U | U | L | L | H |
| Fossey, 2019 | L | U | U | H | L | H |
| Gili, 2020 | U | L | L | H | L | H |
| Gonzalez, 2014 | U | U | U | L | L | H |
| Haringsma, 2006 | U | U | U | L | L | H |
| Hornung, 1999 | U | U | U | L | L | H |
| Hovhannisyan, 2020 | L | L | L | U | L | H |
| Hunkeler, 2012 | L | U | L | L | L | H |
| Hussain, 2017 | U | U | U | U | L | U |
| Jensen, 2019 | L | L | L | H | L | L |
| Jonkers, 2012 | L | U | L | H | L | H |
| Katon, 2001 | L | U | L | U | L | L |
| Kavitha, 2022 | L | L | U | U | L | U |
| Kessing, 2013 | L | H | H | H | L | H |
| Lara-Cabrera, 2016 | L | H | U | H | L | H |
| Lara-Cabrera, 2016 | L | U | U | H | L | H |
| Luciano, 2022 | U | U | U | U | L | U |
| Ludman, 2007 | L | H | L | L | L | H |
| Ludman, 2013 | L | U | U | U | L | H |
| Mishra, 2017 | U | U | U | L | L | H |
| Mueser, 2022 | U | U | U | U | L | U |
| Muralidharan, 2019 | U | U | L | L | L | H |
| Petzold, 2019 | L | U | L | H | L | U |
| Raya-Tena, 2021 | L | U | L | U | L | U |
| Sarabi, 2021 | L | U | L | U | L | U |
| Sato, 2012 | U | U | U | L | L | H |
| Shin, 2002 | U | U | U | U | L | H |
| Simon, 2006 | U | L | L | H | L | H |
| Sousa, 2013 | L | U | H | L | L | H |
| Steigman, 2014 | L | L | L | U | U | U |
| Valencia, 2007 | U | U | L | U | L | H |
| Van Dijk, 2013 | U | L | U | L | L | H |
| Vera, 2010 | L | L | L | L | L | H |
| Zale, 2021 | U | L | U | U | L | H |
