# Supplementary material for: Therapeutic patient education for severe mental disorders: A systematic review
Source: Glob Ment Health (Camb). 2024 Oct 10;11:e78. doi: 10.1017/gmh.2024.68 (PMC11504939; doi:10.1017/gmh.2024.68)
Supplement: Peer review history

# Editor report: Recommendation: Therapeutic patient education for severe mental disorders: A systematic review — R0/PR2

**DOI:** 10.1017/gmh.2024.68.pr2

**Affiliations:** Centre for Rural Health, University of KwaZulu-Natal, South Africa

No accompanying comment.

# Editor report: Decision: Therapeutic patient education for severe mental disorders: A systematic review — R0/PR3

**DOI:** 10.1017/gmh.2024.68.pr3

**Affiliations:** Johns Hopkins University Bloomberg School of Public Health, United States

No accompanying comment.

# Editor report: Recommendation: Therapeutic patient education for severe mental disorders: A systematic review — R1/PR5

**DOI:** 10.1017/gmh.2024.68.pr5

**Affiliations:** Centre for Rural Health, University of KwaZulu-Natal, South Africa

No accompanying comment.

# Editor report: Decision: Therapeutic patient education for severe mental disorders: A systematic review — R1/PR6

**DOI:** 10.1017/gmh.2024.68.pr6

**Affiliations:** Johns Hopkins University Bloomberg School of Public Health, United States

No accompanying comment.
